# Preliminary population genetic analysis of 12 X-STR loci in a sample from Türkiye: allele frequencies, haplotype diversity, and linkage disequilibrium

**DOI:** 10.55730/1300-0144.6221

**Published:** 2026-04-12

**Authors:** Hızır ASLIYÜKSEK, Esra GÜZEL TANOĞLU, Abdulkadir CAN, Muhammed Fevzi ESEN, Yusuf ATAN

**Affiliations:** 1 Council of Forensic Medicine, Ministry of Justice, İstanbul, Turkiye; 2 Department of Molecular Biology and Genetics, Hamidiye Institute of Health Sciences, University of Health Sciences, İstanbul, Turkiye; 3 Department of Health Information Systems, Hamidiye Institute of Health Sciences, University of Health Sciences, İstanbul, Turkiye; 4 Department of Forensic Medicine, Faculty of Medicine, Bilecik Seyh Edebali University, Bilecik, Turkiye

**Keywords:** X chromosome, short tandem repeats, Turkish population, forensic genetics, haplotype frequency, allele frequency

## Abstract

**Background/aim:**

X-chromosomal short tandem repeats (X-STRs) are informative genetic markers in kinship testing and specific forensic contexts, particularly when standard autosomal approaches are less informative. This study aimed to provide preliminary allele frequency, haplotype diversity, and linkage disequilibrium (LD) data for 12 X-STR loci in a sample from Türkiye.

**Materials and methods:**

A total of 136 unrelated individuals from Türkiye were genotyped for 12 X-STR loci. Inferential analyses of X-linked loci require diploid genotypes; therefore, allele frequencies, Hardy–Weinberg equilibrium (HWE), LD, and regional comparisons were based primarily on female samples (n = 115), whereas male haplotypes (n = 21) were summarized descriptively only. Female regional subgroup sizes were uneven (southeast Anatolia, n = 39; Marmara, n = 22; Central Anatolia, n = 14; Mediterranean, n = 13; Black Sea, n = 10; Aegean, n = 9; eastern Anatolia, n = 8). Forensic parameters, HWE exact tests, and pooled and region-stratified LD analyses were calculated using Bonferroni-adjusted thresholds; findings were interpreted cautiously given the modest overall sample size and subgroup imbalance.

**Results:**

All loci were polymorphic, and locus-level forensic efficiency measures were generally high, with DXS10135 showing the highest informativeness in this dataset. In the female sample, five loci deviated from HWE after Bonferroni correction. In pooled LD analysis, only two of 66 locus pairs remained significant after correction (DXS10146–DXS10134 and DXS8378–DXS10103), whereas no region-specific LD signal remained significant after multiple-testing adjustment. Male haplotype diversity estimates were high across linkage groups, but these estimates were based on a small male subset and should be considered exploratory.

**Conclusion:**

This study provides a preliminary X-STR dataset for a sample from Türkiye and suggests that the analyzed panel is informative at the locus level. However, the modest sample size, very limited male subset, uneven regional distribution, and genotype-handling assumptions restrict population-level inference and generalizability. The data may be useful as an initial local reference resource, but broader and more balanced sampling is required before making strong conclusions about national allele distributions, regional structure, or routine forensic implementation.

## 1. Introduction

Short tandem repeats (STRs) are widely used as genetic markers in forensic genetics and kinship testing because of their high polymorphism and interpopulation variability [1]. X-chromosomal STRs (X-STRs) are particularly informative in deficiency cases and complex kinship analyses because of their characteristic inheritance pattern, especially in pedigrees involving female offspring [2]. They have therefore become increasingly relevant in both forensic genetics and population-based studies of genetic variation [3,4]. Beyond their scientific relevance, X-STRs play a critical role in forensic applications, particularly in the identification of individuals who have died in mass disasters such as earthquakes, fires, and floods, as well as in armed conflicts and terrorist attacks [5]. In complex genetic cases in which autosomal STR analysis yields inconclusive results, X-STR markers have demonstrated the capacity to provide highly informative and reliable data. Their utility is especially pronounced in scenarios involving maternal access but incomplete paternal information, such as kinship analyses across second- or third-generation relationships or in the investigation of complex familial relationships and forensic casework [6].

However, the forensic and population genetic value of X-STR data depends on the availability of population-specific allele and haplotype frequencies interpreted in light of linkage, linkage disequilibrium, and possible population substructure [4–6]. Because these characteristics may differ across populations, locally generated reference data remain essential [7].

Although X-STR studies from many populations have been published, available data from Türkiye remain limited. In prior population studies from Türkiye, the geographic origin of the samples has rarely been reported in detail, and the regional diversity across the country has not been sufficiently represented. Accordingly, the current study offers one of the most geographically diverse sample sets to date among published studies investigating 12 X-STR loci in the Turkish population [8–10].

The present study was designed as a preliminary population dataset rather than a definitive national reference panel. Accordingly, the objectives of this study were to describe allele frequencies, locus-wise forensic parameters, haplotype diversity, and linkage disequilibrium patterns of 12 X-STR loci in a geographically diverse but modestly sized sample from Türkiye and to discuss these results with appropriate caution regarding sampling limitations and generalizability.

## 2. Materials and methods

### 2.1. Samples

A total of 136 unrelated individuals (115 females and 21 males) who were sampled through the Republic of Türkiye Ministry of Justice, Forensic Medicine Biology Specialization Department, were included.

Participants were reported to originate from seven geographic regions of Türkiye. For female-based population analyses, the regional distribution was as follows: southeast Anatolia (n = 39), Marmara (n = 22), Central Anatolia (n = 14), Mediterranean (n = 13), Black Sea (n = 10), Aegean (n = 9), and eastern Anatolia (n = 8). All samples were genotyped for 12 X-STR loci (DXS10103, DXS8378, DXS7132, DXS10134, DXS10074, DXS10101, DXS10135, DXS7423, DXS10146, DXS10079, HPRTB, and DXS10148). DNA was extracted using EZ1&2 DNA Investigator Kit (Qiagen, Germany) according to the manucfacturer’s protocol, and alleles were recorded by repeat number. Female samples contributed two alleles per locus, and male samples contributed one allele per locus, consistent with X-chromosome hemizygosity in males.

In a small number of female observations, only a single allele call was available at a locus in the finalized dataset. For consistency of downstream summary analyses, these observations were retained as apparent homozygotes. Because this approach may bias allele frequencies, heterozygosity estimates, and Hardy–Weinberg equilibrium (HWE) testing if some single-allele calls reflect technical dropout rather than true homozygosity [11], all findings potentially affected by this decision were interpreted conservatively, and this issue was explicitly treated as a study limitation.

### 2.2. Typing of 12 X-STR loci using the Investigator Argus X-12 kit

In accordance with International Society for Forensic Genetics (ISFG) guidance and prior validation studies [6,9], the loci were grouped into four linkage groups (LGs): LG1 (DXS10148, DXS10135, DXS8378), LG2 (DXS7132, DXS10079, DXS10074), LG3 (DXS10103, HPRTB, DXS10101), and LG4 (DXS10146, DXS10134, DXS7423) [12–15]. Genotyping was performed using the Investigator Argus X-12 kit (Qiagen, Hilden, Germany) according to the manufacturer’s instructions, and polymerase chain reaction products were analyzed using an ABI Genetic Analyzer (Applied Biosystems, Foster City, CA, USA) [16].

### 2.3. Statistical analysis

Allele frequencies and female-based genotype statistics were calculated from the female subset because HWE and genotype-based linkage disequilibrium (LD) testing for X-STRs require diploid data. Standard forensic parameters were calculated for each locus, including observed heterozygosity (HO), expected heterozygosity (HE), polymorphism information content (PIC), power of discrimination for females (PDF) and males (PDM), and mean exclusion chance (MEC). MEC was calculated using the Desmarais method for duos and the Krüger method for deficiency cases. Male data were not used for inferential LD or HWE analyses because of hemizygosity; instead, male haplotypes were summarized descriptively within each linkage group.

Male haplotype frequencies (n = 21) and Nei’s h were therefore treated as exploratory descriptive measures only and were not interpreted as stable population estimates [17]. HWE was assessed only in females (n = 115) using exact tests. Because multiple loci and locus pairs were tested, Bonferroni correction was applied to control the family-wise type I error rate. It is nevertheless acknowledged that Bonferroni correction is conservative, particularly in small samples and in settings with correlated tests, and may increase type II error; therefore, nonsignificant findings were not interpreted as proof of equilibrium or independence.

The significance threshold for HWE was set at α_HWE_ = 0.05/12 = 0.0042. Regional haplotype comparisons were also based on the female subset and were interpreted as exploratory because subgroup sizes were small and uneven. In each linkage group, multilocus haplotypes were generated from the two alleles at each locus, with locus-wise alleles sorted before concatenation. Pairwise regional differentiation was assessed using the exact test of population differentiation with 10,000 permutations, and AMOVA fixation index (FST) was used as a descriptive measure of between-region differentiation [18]. Following conventional interpretation, FST ≤ 0.01 was considered negligible, 0.01 < FST ≤ 0.050 low, 0.05 < FST ≤ 0.150 moderate, and FST > 0.15 high [18]. Linkage disequilibrium was evaluated in all unrelated female samples. For pairs within the same linkage group, multilocus haplotypes were inferred using an EM algorithm and tested with exact or Monte Carlo haplotype contingency tests; for pairs from different linkage groups, LD was evaluated using genotype (diplotype) contingency tables, with Fisher’s exact test applied to 2 × 2 tables and Pearson’s chi-square test otherwise.

For pooled LD analysis, all 66 pairwise locus combinations were tested in the female sample, yielding a Bonferroni-adjusted threshold of α = 0.00076. Because this threshold is stringent relative to the available sample size, the corrected results were interpreted as minimizing false positives at the cost of reduced power to detect weak or moderate associations.

For region-stratified LD analysis, the same approach was applied separately within each of the seven female regional subgroups (462 tests in total), with a Bonferroni-adjusted threshold of α = 0.00011. Given the limited numbers in several strata, these analyses were considered exploratory and mainly descriptive. Analyses were conducted using R v4.2.3 (packages: HardyWeinberg, pegas, genetics) and Python v3.12.7 (libraries: pandas, numpy, scipy). Arlequin v3.5.2.2 was used for exact tests of population differentiation, AMOVA, and FST estimation [19].

## 3. Results and discussion

### 3.1. Allele frequencies and genetic diversity

All 12 loci were polymorphic in the analyzed dataset, with HO ranging from 0.617 (HPRTB) to 0.887 (DXS10146 and DXS10134) and HE ranging from 0.695 to 0.933. Among the analyzed loci, DXS10135 showed the highest genetic diversity (HE = 0.933), indicating that it was the most informative single marker in this sample. This finding is broadly consistent with previous reports from other populations in which DXS10135 was also identified as one of the most polymorphic and informative X-STR loci, including studies from Northern China, Iraqi Sorani, Namibian, Manchu, Xinjiang Mongolian, and Ecuadorian populations [16,20–23]. Similar findings have also been reported in Northern Thailand and other population datasets, where DXS10135 showed high power of discrimination, high polymorphism information content, and strong exclusion capacity [24,25]. Although direct comparisons across studies should be interpreted cautiously because of differences in sample size, population history, and analytical approach, the repeated observation of high informativeness at DXS10135 supports the relevance of this locus for X-STR panels across diverse populations [16,20–23].

HWE was assessed only in females (n = 115), as appropriate for diploid X-chromosomal genotype data. After Bonferroni correction (α = 0.0042), five loci (DXS8378, DXS10103, HPRTB, DXS7423, and DXS10074) remained significant. These deviations should be interpreted with caution. In a dataset of this size, departures from HWE may reflect stochastic variation, regional substructure, uneven sampling across subgroups, null alleles, technical issues, or the handling of single-allele female observations as apparent homozygotes. Therefore, the HWE results should not be interpreted as definitive evidence of biological disequilibrium at these loci but rather as signals that warrant cautious interpretation and further evaluation in larger and more balanced samples.

### 3.2. Forensic efficiency and genetic diversity of X-STR loci and linkage groups

Forensic efficiency parameters for the 12 X-STR loci demonstrated generally high locus-level informativeness in this dataset (Table 1). PIC exceeded 0.60 for all loci, with the highest value observed for DXS10135 (0.928). PDF ranged from 0.837 to 0.984, while PDM ranged from 0.580 to 0.925. DXS10135 again showed the highest overall discriminatory capacity (PDF = 0.984; PDM = 0.925), followed by DXS10148, DXS10146, and DXS10101. By contrast, DXS7423 and DXS8378 showed relatively lower PIC and discrimination values. These results suggest that the panel is informative at the locus level in the present sample, although such estimates should be interpreted as sample-based rather than as definitive national population parameters.

The pattern observed for DXS10135 is in line with previous studies reporting this locus as one of the most informative X-STRs in different populations. In Northern Thailand, DXS10135 showed the highest polymorphism, discrimination power, and mean exclusion chance [24]. Similarly, in Ecuadorian and Northern Chinese populations, DXS10135 was reported among the most informative loci with respect to gene diversity, PIC, and discrimination capacity [23,25]. The concordance of this locus-level pattern across populations strengthens the interpretation that DXS10135 is consistently highly polymorphic, even though the present dataset remains limited in size and does not permit strong population-level generalization.

Male haplotype diversity estimates were high across all linkage groups (LG1: 0.9912; LG2: 0.9842; LG3: 0.9863; LG4: 0.9748). These findings suggest substantial haplotypic diversity within the observed male subset. However, because these estimates were derived from only 21 males, they should be regarded as exploratory descriptive measures rather than stable population estimates. The male haplotype results are included to describe the observed structure of the dataset and not to support firm conclusions regarding haplotype frequencies in the broader Turkish population.

### 3.3. Linkage disequilibrium analysis

Pairwise LD analysis was performed in female samples in accordance with standard X-STR practice. In Table 2, among the 66 tested locus pairs, only two remained significant after Bonferroni correction: DXS10146–DXS10134 within LG4 (p = 0.0004) and DXS8378–DXS10103 across linkage groups (p = 0.00043). The signal observed for DXS10146–DXS10134 is biologically plausible because both loci belong to the same linkage group and are physically close on the chromosome. This finding is consistent with previous literature. In a Brazilian population, five marker pairs remained significant after Bonferroni correction, including DXS10146–DXS10134 [26]. Likewise, studies from Germany have emphasized the close molecular relationship and low recombination pattern among LG4 markers, particularly DXS10146 and DXS10134, supporting the forensic relevance of haplotype-based treatment for this region [13]. In Korean datasets, closely linked marker trios involving DXS10146–DXS10134–DXS7423 and DXS10148–DXS10135–DXS8378 also showed high haplotype diversity and forensic utility [27].

By contrast, the significant intergroup association between DXS8378 and DXS10103 should be interpreted much more cautiously. Because these loci are not physically linked, the observed signal is unlikely to represent true physical linkage. In the context of a modest sample size, such a result may arise from sampling fluctuation, population substructure, sparse contingency tables, or residual statistical noise. Therefore, although the signal is statistically notable in the present dataset, it should not be interpreted as evidence of stable biological linkage between these loci.

Most locus pairs did not remain significant after multiple-testing correction. However, the absence of significant LD should not be overinterpreted as proof of complete marker independence. Bonferroni correction is conservative, especially in a study of modest size, and may increase the risk of false-negative results. Thus, the present findings suggest that strong pairwise LD signals were uncommon in this dataset, but they do not exclude the possibility of weaker associations that could become apparent in larger and better-powered samples.

### 3.4. Regional comparisons and population structure

As shown in Table 3, regional comparisons of X-STR haplotypes did not reveal statistically significant pairwise differences within linkage groups. At face value, this pattern may suggest limited detectable interregional differentiation in the current dataset. However, such an interpretation must remain cautious because regional subgroup sizes were highly uneven and, in several cases, small (e.g., Black Sea n = 10, Aegean n = 9, eastern Anatolia n = 8). Under these conditions, nonsignificant results may reflect limited statistical power rather than true homogeneity.

AMOVA yielded a multilocus FST of 0.0306 (95% CI: 0.0278–0.0338), with permutation testing indicating statistical significance (p = 0.0146). According to the predefined classification, this corresponds to low but detectable differentiation [18]. This result suggests that some level of between-region variation may be present in the dataset. However, because the sample is modest and regionally imbalanced, this finding should not be expanded into strong claims about the structure of the Turkish population as a whole. Türkiye has a complex demographic history and potential admixture across regions, and the current dataset is not sufficiently large or balanced to resolve such structure robustly. Accordingly, the regional analyses are best regarded as exploratory summaries that may guide future work rather than as definitive evidence for either homogeneity or stratification.

### 3.5. Regional linkage disequilibrium analysis

The region-stratified LD analysis comprised 462 tests (66 locus pairs across seven female regional subgroups) as presented in Table 4. After Bonferroni correction (α = 0.00011), no region-specific LD signal remained significant. Nominal significance (uncorrected p < 0.05) was observed in 12 pairwise comparisons, with the smallest p value detected for DXS10103–DXS10079 in southeast Anatolia (p = 0.00278). None of these findings survived correction for multiple testing.

This overall pattern should again be interpreted cautiously. In regions with very small subgroup sizes, the lack of significant LD after correction may simply reflect insufficient power. Conversely, isolated nominal associations may arise stochastically in sparse tables. The absence of corrected significance therefore does not prove complete regional independence of all markers. Rather, it indicates that no strong and reproducible region-specific LD signal could be demonstrated within the limits of the present sample.

Taken together, the regional and pooled LD results suggest that the analyzed X-STR panel shows largely limited detectable pairwise association in this dataset, with one biologically plausible intralinkage group signal (DXS10146–DXS10134) and one statistically significant but biologically less straightforward intergroup signal (DXS8378–DXS10103). These results are compatible with prior studies showing that some physically linked X-STR loci may exhibit LD, while many other pairwise relationships remain weak or sample-dependent [13,26,27]. However, larger datasets with more balanced regional recruitment are needed before broader conclusions can be drawn regarding LD structure and regional population patterns in Türkiye.

## 4. Conclusion

This study presents preliminary population genetic data for 12 X-STR loci in a sample from Türkiye, including allele frequencies, locus-wise forensic parameters, haplotype summaries, and linkage disequilibrium patterns across seven geographic regions. All analyzed loci were polymorphic, and several loci, particularly DXS10135, DXS10148, DXS10146, and DXS10134, showed high locus-level informativeness in this dataset. In female samples, five loci (DXS8378, DXS10103, HPRTB, DXS7423, and DXS10074) deviated from Hardy–Weinberg equilibrium after Bonferroni correction. These findings warrant cautious interpretation and continued quality assessment; however, they do not by themselves negate the utility of the panel.

In pooled LD analysis, only two locus pairs remained significant after multiple-testing correction: DXS10146–DXS10134 within LG4 and DXS8378–DXS10103 across linkage groups. The intragroup signal for DXS10146–DXS10134 is consistent with previous evidence that closely linked X-STRs may show nonrandom association, whereas the intergroup finding should be interpreted more cautiously because it may reflect sampling variation, substructure, or other nonbiological sources rather than true physical linkage. No region-specific LD signal remained significant after Bonferroni correction; however, given the modest sample size and the small size of several regional subgroups, this should not be interpreted as definitive evidence of complete marker independence across all regions.

Recent studies have significantly expanded the global understanding of X-STR diversity and its forensic applications. Gusmão et al. compiled and analyzed a large dataset of over 23,000 X-chromosomal haplotypes from worldwide populations, including more than 10,000 newly generated haplotypes, and highlighted the importance of metapopulation approaches and accurate mutation rate estimates for the reliable interpretation of X-STR profiles across different genetic backgrounds [28]. Similarly, Pilav et al. reported allele and haplotype frequencies for the 12 X-STR loci of the Investigator Argus X-12 kit (Qiagen, Hilden, Germany) in the Bosnia and Herzegovina population, demonstrating high polymorphism and forensic efficiency while revealing subtle genetic differences even among geographically neighboring European groups [29]. In East Asia, Lee et al. analyzed 12 X-STRs in 427 Korean males and observed distinct haplotype patterns that clustered with other East Asian populations but differed markedly from European and Middle Eastern groups. These recent studies underscore the continued need for population-specific reference data, as allele and haplotype frequencies as well as linkage patterns can vary considerably across populations [30]. In this context, data from the Turkish population remain limited and geographically underrepresented, further justifying the contribution of the present study through its broader regional sampling.

This study has some limitations that restrict population-level inference. The overall sample size was modest, the male subset was very small (n = 21), and regional sampling was clearly imbalanced, with some subgroups represented by fewer than 10–14 individuals. As a result, male haplotype diversity estimates should be regarded as descriptive and exploratory rather than stable population parameters, and regional comparisons should not be overinterpreted as evidence of nationwide homogeneity. In addition, retaining single-allele female observations as apparent homozygotes may have influenced allele frequency estimates, heterozygosity, and HWE results if any such calls reflected technical dropout rather than true homozygosity. Finally, although Bonferroni correction appropriately reduced the risk of false-positive findings, it likely increased the risk of false-negative results, particularly in LD and region-stratified analyses.

Accordingly, the present findings should be viewed as an initial local dataset rather than a definitive national reference resource. Within these constraints, the 12-locus panel appears informative at the locus level and may contribute useful preliminary data for future forensic and population-genetic work in Türkiye. Larger studies with more balanced regional recruitment, substantially expanded male sampling, and sensitivity analyses based on original genotype-call uncertainty are needed before stronger conclusions can be drawn regarding regional structure, LD stability, or routine nationwide reference use.

## Figures and Tables

**Table 1:** Forensic efficiency parameters of 12 X-STR loci across four linkage groups.

Linkage group	Gene diversity (h) (group-level, male haplotype)*	Locus	Number of alleles	PD_F_	PD_M_	PIC	MEC (Krüger)	MEC (Desmarais duo)
LG1	0.9912							
		DXS10148	32	0.975	0.844	0.897	0.821	0.904
		DXS10135	25	0.984	0.925	0.928	0.870	0.932
		DXS8378	9	0.837	0.684	0.641	0.497	0.698
LG2	0.9842							
		DXS7132	7	0.881	0.666	0.675	0.533	0.717
		DXS10079	12	0.940	0.802	0.790	0.670	0.814
		DXS10074	13	0.944	0.811	0.812	0.699	0.833
LG3	0.9863							
		DXS10103	8	0.888	0.720	0.691	0.550	0.724
		HPRTB	7	0.879	0.748	0.673	0.532	0.719
		DXS10101	18	0.973	0.831	0.885	0.802	0.894
LG4	0.9748							
		DXS10146	22	0.978	0.870	0.898	0.822	0.905
		DXS10134	20	0.965	0.793	0.855	0.760	0.868
		DXS7423	7	0.842	0.580	0.638	0.494	0.694
Combined								

* Male-derived gene diversity estimates are exploratory because of the small sample size (n = 21).

LG: linkage group; PD_F_: power of discrimination for females; PD_M_: power of discrimination for males; PIC: polymorphism information content; MEC: mean exclusion chance.

**Table 2:** Linkage disequilibrium (LD) for 12 X-STR loci across four linkage groups.

Linkage group	Locus1	Locus2	p	Linkage group	Locus1	Locus2	p
1/1	DXS10148	DXS10135	0.084	2/3	DXS7132	HPRTB	0.208
1/1	DXS10148	DXS8378	0.998	2/3	DXS7132	DXS10101	0.603
1/2	DXS10148	DXS7132	0.080	2/4	DXS7132	DXS10146	0.291
1/2	DXS10148	DXS10079	0.730	2/4	DXS7132	DXS10134	0.002
1/2	DXS10148	DXS10074	0.320	2/4	DXS7132	DXS7423	0.660
1/3	DXS10148	DXS10103	0.881	2/2	DXS10079	DXS10074	0.019
1/3	DXS10148	HPRTB	0.562	2/3	DXS10079	DXS10103	0.978
1/3	DXS10148	DXS10101	0.432	2/3	DXS10079	HPRTB	0.954
1/4	DXS10148	DXS10146	0.714	2/3	DXS10079	DXS10101	0.891
1/4	DXS10148	DXS10134	0.007	2/4	DXS10079	DXS10146	0.336
1/4	DXS10148	DXS7423	0.606	2/4	DXS10079	DXS10134	0.531
1/1	DXS10135	DXS8378	0.002	2/4	DXS10079	DXS7423	0.946
1/2	DXS10135	DXS7132	0.538	2/3	DXS10074	DXS10103	0.011
1/2	DXS10135	DXS10079	0.201	2/3	DXS10074	HPRTB	0.684
1/2	DXS10135	DXS10074	0.340	2/3	DXS10074	DXS10101	0.222
1/3	DXS10135	DXS10103	0.342	2/4	DXS10074	DXS10146	0.005
1/3	DXS10135	HPRTB	0.459	2/4	DXS10074	DXS10134	0.676
1/3	DXS10135	DXS10101	0.119	2/4	DXS10074	DXS7423	0.147
1/4	DXS10135	DXS10146	0.479	3/3	DXS10103	HPRTB	0.896
1/4	DXS10135	DXS10134	0.996	3/3	DXS10103	DXS10101	0.044
1/4	DXS10135	DXS7423	0.360	3/4	DXS10103	DXS10146	0.060
1/2	DXS8378	DXS7132	0.992	3/4	DXS10103	DXS10134	0.214
1/2	DXS8378	DXS10079	0.053	3/4	DXS10103	DXS7423	0.788
1/2	DXS8378	DXS10074	0.071	3/3	HPRTB	DXS10101	0.257
1/3	DXS8378	DXS10103	0.000	3/4	HPRTB	DXS10146	0.686
1/3	DXS8378	HPRTB	0.623	3/4	HPRTB	DXS10134	0.120
1/3	DXS8378	DXS10101	0.930	3/4	HPRTB	DXS7423	0.293
1/4	DXS8378	DXS10146	0.169	3/4	DXS10101	DXS10146	0.077
1/4	DXS8378	DXS10134	0.071	3/4	DXS10101	DXS10134	0.832
1/4	DXS8378	DXS7423	0.033	3/4	DXS10101	DXS7423	0.191
2/2	DXS7132	DXS10079	0.012	4/4	DXS10146	DXS10134	0.000
2/2	DXS7132	DXS10074	0.917	4/4	DXS10146	DXS7423	0.150
2/3	DXS7132	DXS10103	0.934	4/4	DXS10134	DXS7423	0.052

For locus pairs within the same linkage group (intragroup), LD was tested in females using EM-inferred haplotypes and exact or Monte Carlo evaluation of haplotype contingency tables. For locus pairs from different linkage groups (intergroup), LD was tested in females using genotype (diplotype) contingency tables, with Fisher’s exact test applied to 2 × 2 tables and Pearson’s χ^2^ test applied to larger tables. p-values were obtained from Fisher’s exact test or Pearson’s chi-square test, with Bonferroni correction as described in Section 2.

**Table 3:** Interregional genetic differentiation analysis for X-STR linkage groups.

Linkage group	Most differentiated region pair	p-value (σ)	Least differentiated region pair	p-value (σ)	Genetic interpretation
LG1	Black Sea vs. Central Anatolia	0.358 (0.043)	Marmara vs. EasternAnatolia	0.540 (0.038)	Descriptive contrast only; no significant differentiation detected
LG2	Black Sea vs. Marmara	0.089 (0.049)	SE Anatolia vs. Mediterranean	0.841 (0.024)	Largest observed contrast in this table, but still nonsignificant and exploratory. Possible indication of coastal homogeneity vs. inland variation
LG3	Marmara vs. Central Anatolia	0.226 (0.034)	Black Sea vs. Aegean	0.411 (0.048)	Little detectable differentiation in this sample. Possibly, uniform conservation across regions
LG4	Black Sea vs. Mediterranean	0.565 (0.042)	SE Anatolia vs. Aegean	0.412 (0.026)	Descriptive pattern only; no evidence for firm regional inference but possible interregional stability

LG: linkage group; vs.: versus; p-values were obtained from exact tests of population differentiation.

**Table 4:** Regional summary of nominal linkage disequilibrium signals.

Region	Sample (n)	Tests (n)	Nominal hits (%)	Smallest p	Most notable pair (lowest p)
Southeast Anatolia	39	66	7 (10.6)	0.002	DXS10103–DXS10079
Marmara	22	66	5 (7.6)	0.043	DXS10103–HPRTB
Black Sea	10	66	0 (0)	0.066	DXS7132–DXS10079
Aegean	9	66	0 (0)	0.101	DXS10074–DXS10079
Mediterranean	13	66	0 (0)	0.095	DXS10074–DXS10079
Central Anatolia	14	66	0 (0)	0.133	DXS10134–DXS10146
Eastern Anatolia	8	66	0 (0)	0.104	DXS10103–DXS10101

LD results in small regions (n < 15) are exploratory because of limited power. The nominal hits variable is defined as the proportion of locus pairs with uncorrected p < 0.05, which are interpreted as nonsignificant after Bonferroni adjustment (α = 0.00011). p-values were obtained from the linkage disequilibrium testing procedure described in Section 2.
